# The Scalable Fuzzy Inference-Based Ensemble Method for Sentiment Analysis

**DOI:** 10.1155/2022/5186144

**Published:** 2022-09-28

**Authors:** Yunus Emre Isikdemir, Hasan Serhan Yavuz

**Affiliations:** Eskisehir Osmangazi University, Electrical and Electronics Engineering Department, Eskisehir 26480, Turkey

## Abstract

Internet environments such as social networks, news sites, and blogs are the platforms where people can share their ideas and opinions. Many people share their comments instantly on the internet, which results in creating large volumes of entries. It is important for institutions and organizations to analyze this big data in an efficient and rapid manner to produce summary information about the feelings or opinions of individuals. In this study, we propose a scalable framework that makes sentiment classification by evaluating the compound probability scores of the most widely used methods in sentiment analysis through a fuzzy inference mechanism in an ensemble manner. The designed fuzzy inference system makes the sentiment estimation by evaluating the compound scores of valance aware dictionary, word embedding, and count vectorization processes. The difference of the proposed method from the classical ensemble methods is that it allows weighting of base learners and combines the strengths of each algorithm through fuzzy rules. The sentiment estimation process from text data can be managed either as a 2-class (positive and negative) or as a 3-class (positive, neutral, and negative) problem. We performed the experimental work on four available tagged social network data sets for both 2-class and 3-class classifications and observed that the proposed method provides improvements in accuracy.

## 1. Introduction

Sentiment analysis has been carried out through some special algorithms designed to determine whether individuals' attitudes towards a particular topic are positive, negative, or neutral from their comments or writings. In this way, valuable information that is extremely important for companies or institutions such as the opinions of customers about brands and products or the satisfaction of individuals can be extracted. Today, due to the widespread use of the internet, texts shared in many online platforms such as social media platforms, shopping sites, news channels, and forums create large amount of data [1]. It is of great importance to analyze this big data, which is constantly changing and is updated, in an efficient and rapid manner.

Sentiment analysis is a subfield of natural language processing. It is operated with machine learning algorithms that are designed to understand and classify text inputs. Sentiment analysis is important for a wide variety of sectors such as determining the tendencies of customers in the business world, understanding student behaviour in the education sector, and detecting the emotions of society in government affairs [2, 3]. Affective computing and sentiment analysis find applications in various companies and scenarios that include emotion analysis as a part of their mission [4]. They have a great potential to enhance the capabilities of recommendation systems or customer relationship management [4]. Due to the fact that the volume of data shared on the internet is continuously increasing, it creates the necessity of performing sentiment analysis on big data effectively for many different application areas ranging from social media [5] to financial markets [6].

Sentiment analysis has a wide range of applications and can be divided into three categories based on the type of the methods applied such as dictionary-based, machine-learning-based, and deep-learning-based [3]. Although each category has its own advantages and disadvantages, researchers face significant challenges such as dealing with context, sarcasm, slang, lexical, and syntactic ambiguities in writing. Sarcasm is an ironic or satirical description softened by humor. In general, people use it to exaggerate by saying the opposite of the truth while expressing their feelings in comments they enter on social media platforms, blogs, shopping sites, etc. [7, 8]. Sarcasm detection is relevant to sentiment analysis to accurately recognize users' opinion or orientation on a specific topic, which could be a service, product, person, organization, or event [9]. Sarcasm detection plays an important role in improving the performance of natural language processing applications [10, 11].

Social media usage has been increasing rapidly that results in a new form of written text called microtext [12]. Since there are no standard rules for writing across multiple platforms, people use short messages that may also include misspelling with unconventional grammar and style. Microtext normalization is considered as the recovery of the intended word in an observed nonstandard word [13]. Satapathy et al. [14] classified approaches for microtext normalization into three categories, namely, the syntax-based approach, probability-based approach, and phonetic-based approach. In [15], they showed that incorporating deep learning models into a microtext normalization module helps improve sentiment analysis.

The semantic orientation of an opinion shows whether it is positive, negative, or neutral. In sentiment analysis, there are two types of techniques for the semantic orientation-based approach, namely, the corpus-based approach and dictionary-based approach [16]. In the corpus-based approach, the polarity value is calculated depending on the co-occurrences of the term with other positive or negative seed words in the corpus. Dictionary-based approaches, on the other hand, use predeveloped polarity lexicons such as WordNet [17], SentiWordNet [18], and SenticNet [19]. The semantic orientation-based approaches primarily extract sentiment-rich features from the unstructured text based on corpus or dictionary. Then, overall polarity of the document is resolved by aggregating the semantic orientations of all features [16]. Althoughmost studies in this field have primarily used English, there are many applications of sentiment analysis in other languages [20–24]. New trends on neurosymbolic artificial intelligence for explainable sentiment analysis include unsupervised, reproducible, interpretable, and explainable frameworks such as SenticNet7 [19] and OntoSenticNet2 [25].

Sentiment analysis studies still remain popular because it is possible to integrate different knowledge-based representations into sentiment analysis systems to enhance reasoning. Fuzzy set theory is known to be quite successful in modeling and managing uncertainty and linguistic descriptions mathematically [26]. Therefore, it is possible to apply fuzzy sets and fuzzy reasoning to express sentiment's polarity [27]. Yu et al. [28] added fuzzy reasoning to the neural network classifier model to establish a multimodal and multiscale emotion-enhanced inference. Vashishtha and Susan [29] proposed a neuro-fuzzy network that incorporates inputs from multiple lexicons to perform sentiment analysis of social media posts, called MultiLexANFIS. AL-Deen et al. [30] proposed a sentiment classification method based on the fuzzy rule-based system with the crow search algorithm and obtained high accuracy relative to existing approaches. Yan et al. [31] proposed an emotion-enhanced reference model that includes fuzzy reasoning. These studies illustrate that the fuzzy logic can be utilized in many different subunits of sentiment analysis such as sentence level learning, decision-making, classifier design, and consensus improvement [32].

For learning based strategies, it is well known that deep learning methods give more effective results in the presence of large training data [33]. However, the model fitting process takes a long time in the case of big data since the volume of the data is very large. Moreover, parameter optimization for deeper models takes too long, which is not reasonable in terms of both memory and time. For such big data, it is more efficient to use scalable models. Scalability is known as the ability of a system to handle an increasing amount of work. Developing a methodology that can work efficiently in all fields is still a major challenge for researchers [34].

In this article, we propose a method that considers the data from different perspectives to solve the text-to-sentiment classification problem. In the proposed method, the most widely used data processing methods in sentiment analysis are selected for increasing scalability, and the compound scores are interpreted by a fuzzy inference mechanism. In the designed structure, a word-based inference is made with logistic regression and count vectorization; on the other hand, the relations between words or sequential correlation are learned by examining the bidirectional long-short term memory (LSTM) with word embedding. In addition, the polarity scores of sentences are calculated with the valence aware dictionary and sentiment reasoner (VADER). Overall information is interpreted with a fuzzy rule-based mechanism for the final decision-making. The proposed method can be adapted to different sentiment classification problems in other fields by making minor changes in the fuzzy inference design without requiring additional training in deep learning models.

## 2. Background

Sentiment analysis is generally defined as the use of computational linguistics such as natural language processing or text analysis to extract, identify, and study the subjective information based on customer reviews or survey responses. The main task in sentiment analysis is to classify whether the opinion expressed in a particular text is positive, negative, or neutral. Further tasks include distinguishing emotions such as pleasure, disgust, sadness, anger, fear, or surprise [35]. Emotion is a complex psycho-physiological change that arises from the interaction of the individual's mood with biochemical and environmental influences. Therefore, it has been researched by many disciplines and art forms [36]. Emotional assistance studies aim to discover the underlying reasons behind the emotional expression in texts [37]. The issue of the number and classification of emotions is still challenging because it differs in different languages and cultures [38].

Many studies on sentiment analysis of texts collected from social networks and microblogging websites focus on the classification of texts as either a binary classification problem (positive and negative) or a ternary classification problem (positive, negative, and neutral) [39]. Although it is considered as a ternary classification problem, neutral reviews are often ignored because of their lack of information or their ambiguity in many sentiment analysis problems [40]. Neutrality refers to not supporting either side in a controversy. Valdivia et al. [41] empower neutrality by characterizing the boundary between positive and negative reviews to improve the classification performance. They proposed consensus vote models for detecting and filtering neutrality to improve the sentiment classification. Wang et al. [42] draw attention to ambivalence sentiments. Ambivalence is defined as the state of having mixed feelings or contradictory ideas, so they consider ambivalence sentiments as the mixture of positive and negative comments. In [42], they presented a multilevel sentiment sensing scheme with the strength level tune parameters for analyzing the strength and fine-scale of positive and negative sentiments. They showed that the ambivalence handler increased the overall performance of the algorithms.

The first stage of text classification in conventional sentiment analysis is the conversion of text data into numerical values that machines can understand. Then, the category of data is determined by making statistical inferences. In this context, there are various methods in the literature to convert the text data into numerical format and to categorize them. One of the most popular among these methods is the count vectorization. The count vectorization method basically counts the occurrence of each word in the document and uses this value as a variable in order to predict the target variable [43]. As an alternative to the count vectorization method, the term frequency-inverse document frequency (TFIDF) method uses weightings of the words in the document [44]. In this way, the importance of each word in the document can be determined. Both the count vectorization and TFIDF methods do not consider the relations between words. Word embedding can be used in order to determine similarity and relationship between words representing as a vector in the vector space.

There are many studies on natural language processing for predictive modeling of reviews, text generation, and text classification [45–47]. In this context, machine learning and deep learning approaches are getting more and more popular in this field [48–50]. The most used traditional machine learning approaches used in text classification tasks are the support vector machines (SVMs), logistic regression, and naive Bayes methods [51–53]. These algorithms yield better results with count vectorization and TFIDF vectorization [54]. However, significantly increasing dimensions cause more memory consumption, and it is also difficult to add extra features when using count vectorization and TFIDF vectorization.

For the last decade, deep learning produced very successful results in many application areas such as computer vision, speech recognition, and natural language processing compared to the previously mentioned machine learning methods. Deep neural networks are capable of extracting nonlinear relations from the given data. Traditional neural networks may give poorer results in text classification tasks compared to deep networks because they do not consider sequential correlations as deep networks [55] do. The recurrent neural network (RNN) architecture is capable of dealing sequential data, but the vanishing gradient is a big problem for deeper networks [56]. The vanishing gradient problem is unavoidable for the RNN due to its architecture where weights disappear with backpropagation. Since the weights in each layer are adjusted via chain rules, the gradient values will shrink exponentially when stepped back, and eventually, they will disappear. In order to handle the vanishing gradient problem, gated recurrent units (GRU) and LSTM variants are used [57]. Unlike the traditional sentiment analysis, which estimates the overall sentiment of a particular text, aspect-based sentiment analysis aims to detect the sentiment polarities of different aspects in the same sentence. Chen et al. [58] proposed a model that integrates the graph convolution network and the coattention mechanism to cope with the aspect-based information. Liang et al. [59] proposed an affective knowledge-enhanced graph convolutional network based on SenticNet to leverage affective dependencies of sentences according to the specific aspect. Sentiment analysis may also be applied in live conversations, called conversational sentiment analysis, to improve human-machine interaction. Recently, Li et al. [60] proposed the bidirectional emotional recurrent unit for conservational sentiment analysis. They used a neural tensor block followed by a two-channel classifier to perform context compositionality and sentiment classification.

Fuzzy set theory, or more simply called the fuzzy logic, has been successfully used to describe uncertain situations after being proposed by Lotfi Zadeh [61]. The structure of fuzzy set theory makes it possible to define and use linguistic terms in logical inference. By this way, fuzzy logic models can represent, manipulate, interpret, and use uncertain and imprecise data and information. The fuzzy logic has been applied in many different ways in sentiment analysis. Dragoni and Petrucci proposed a fuzzy-based strategy for multidomain sentiment analysis [62]. They used the fuzzy logic for representing the polarity learned from training sets and integrated this information with further conceptual knowledge. Vashishtha and Susan [63] analyzed the sentiment of social media posts using a nine fuzzy rule-based system to classify the post into three classes: negative, positive, or neutral. They showed that fuzzy reasoning is able to incorporate the positive and negative scores. Madbouly et al. [64] proposed a hybrid model for twitter posts in which the lexicon-based methodology is combined with a fuzzy classification technique to handle language vagueness. Sivakumar and Uyyala [65] applied aspect-based sentiment analysis on mobile phone reviews using the fuzzy logic and LSTM from online shopping sites. Aspect-based sentiment analysis using LSTM with FL adopts the features of the ClausIE framework for splitting long sentences into meaningful small pieces. They showed that the word embedding technique was well suited for aspect-based sentiment analysis.

The type of methodologies in sentiment analysis can be classified into the following three categories: statistical methods, knowledge-based techniques, and hybrid approaches [66]. Statistical methods include machine learning concepts such as latent semantic analysis, bag of words, support vector machines, and deep learning. Knowledge-based techniques classify the text into emotion categories based on the presence of ambiguous emotion words. Some knowledge bases not only include the list of affective words but also assign probable affinities to particular emotions. Hybrid approaches use both machine learning and knowledge-based elements such as semantic networks and ontologies to determine semantics in a subtle manner [67].

In this paper, we propose a scalable fuzzy inference-based sentiment classification framework. Our main objective here is to propose a hybrid mechanism that evaluates the outputs of some effective methods used in sentiment analysis through a fuzzy inference mechanism to increase the efficiency. Under the existence of large volume or big data, the training of a deep learning-based methodology requires high computational power and consumes a plenty of time. In our design, we use the pretrained models of some effective methods as the input and interpret the sentiment decision by means of a fuzzy rule base. The fuzzy rule-based mechanism does not require any new training for continuously growing data or big data. In the following subsections, we first mention some fundamental concepts in sentiment analysis and then present the proposed methodology in the next section.

### 2.1. Text Preprocessing and Feature Extraction

Text preprocessing is a crucial step for sentiment analysis [68]. The process eliminates the noninformative data and transforms the data into a standard form in order to improve the performance of the algorithm. The first step of text preprocessing is text cleaning. The following items are among the most widely used techniques in text cleaning:

- Converting each character to lowercase is a necessary step to prevent some words from being perceived as unique words. For example, “Good” and “good” are considered different words when characters are not converted to lowercase.
- Punctuations and numbers do not contain information so that removing them can increase the accuracy while preventing bias. It also reduces unnecessary memory consumption.
- Tokenization is a process that splits each review into words as tokens. These words will be treated as variables in order to predict the target variable.
- Stop words are the commonly used words in a language such as “a,” “the,” and “as” that do not contain information. They are used for the connection of words in a sentence so that they can be removed.
- Lemmatization is a process that reduces words into stems considering morphological analysis. In this context, SpaCy library [69] might be used for stop word elimination and lemmatization.

Feature extraction is another important step for sentiment analysis from subjective text. In the feature extraction stage, text data are converted into integer tokens that can be processed by machine learning methods. There are various feature extraction techniques such as bag of words, N-grams, TFIDFs, and word embedding [3]. Ahuja et al. [70] presented that TFIDF gives 3-4% higher performance than N-gram features.

The TFIDF vectorization consists of two items that are called term frequency and inverse document frequency. Theterm frequency measures the frequencies of the words in the document, and higher appearances imply significant words. The inverse document frequency measures rare words in the collection. The words which have higher frequencies in the collection mean that words are not representative for the document and that the rare words in the collection are important for this document.

Word2Vec [71] and GloVe [72] are word embedding methods with different approaches from count vectorization and TFIDF vectorization methods. In these methods, each word is represented as a vector instead of their counted or normalized values. Word vectors are assumed to be positioned in a vector space so that words that share common contexts in the sentence are close to each other. After the words are translated into numerical values called features, they can be evaluated by various machine learning methods for the classification. In the following subsections, we briefly present some classical machine learning and deep learning methodologies that are widely used in sentiment analysis.

### 2.2. Some Popular Classical Machine Learning-Based Methods

Machine learning involves how computers can perform a variety of tasks such as decision-making and classification. Commonly used machine learning methods in sentiment analysis are known as naive Bayes, the SVM, and logistic regression.

#### 2.2.1. Naive Bayes

A naive Bayes classifier is a probabilistic classifier that is based on the Bayes theorem [73]. In this method, probability of the outcome is produced by the conditional probability model. Let the instance to be classified be represented by a vector **x**=(*x*_1_,…, *x*_*n*_) with *n* features. The naive Bayes classifier makes the assignment among a possible outcome among *K* classes, *C*_*k*_, according to the following formulation:

(1) $$\begin{array}{c} p\left(C_{k}\;|\;x\right)=\frac{p\left(C_{k}\right)p\left(x\;|\;C_{k}\right)}{p\left(x\right)}\text{.} \end{array}$$

The naive Bayes algorithm can be used as binary or multiclass classification. One advantage of naive Bayes is that it requires a small number of training data to estimate the parameters required for classification.

#### 2.2.2. Support Vector Machines

The SVM aims to determine a linear hyperplane that passes through the middle of the maximum margin between the classes for a two-class data set in the feature space [74]. This separating hyperplane is determined with an optimization problem to maximize the spacing between classes. In terms of the hinge loss, the optimization problem is defined as the following loss minimization:

(2) $$\begin{array}{c} \lambda {\left\|w\right\|}^{2}+\left[\frac{1}{n}\overset{n}{\underset{i=1}{\sum }}\max\left(0,1-y_{i}\left(w^{T}x_{i}-b\right)\right)\right]\text{,} \end{array}$$

where *λ* > 0 is a parameter to determine the trade-off between increasing the margin size and at the same time ensuring that the sample **x**_*i*_ lies on the correct side of the margin. Also, *y*_*i*_=−1 or 1 denotes the class label and **w**^*T*^**x**_*i*_ − *b* is the *i*^th^ output. This problem can be solved in primal form or dual form to find the separating plane. Keen readers may refer to [75] to see the optimization details.

#### 2.2.3. Logistic Regression

Logistic regression is a supervised learning algorithm that is based on the logistic function [76]. The logistic function is an *S*-shaped curve that maps the values between 0 and 1. The following formulation depicts the simplified form of the logistic function:

(3) $$\begin{array}{c} P=\frac{1}{1+e^{-\left(\beta_{0}+\beta_{1}x_{1}\right)}}\text{.} \end{array}$$

Here, *P* describes the probability, *e* is the Euler number, and *β*_0_ and *β*_1_ are the parameters of the model. Logistic regression can be binomial, multinomial, or ordinal. Binomial logistic regression deals with binary situations, i.e., there are only two possible outcomes, 0 or 1. Multinomial logistic regression deals with three or more possible outcome types that are not ordered. Ordinal logistic regression deals with dependent variables that are ordered.

### 2.3. Some Popular Deep Learning-Based Methods

Deep learning is a modern variation of artificial neural networks that concerns with an unbounded number of layers of bounded size that permits an optimized implementation of practical implementations [77]. The capability of handling large and complex data makes deep learning more important for text analytics. Some of the most widely used deep learning-based methodologies in sentiment analysis are presented below.

#### 2.3.1. Simple RNN

**Figure 1:**
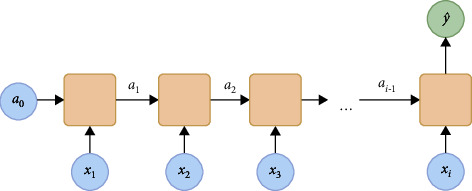
Many to one RNN.

Traditional neural networks have been used to model the classification and regression problems for years. However, there are extra constraints of modeling sequential data. Recurrent neural networks are the sequential based networks that address this problem considering the order of the input [78]. In contrast to traditional networks, the RNN takes input from current time steps as well as previous time steps as depicted in Figure 1. In this way, time dependency can be handled.

This type of network is more suitable for modeling sequential data such as time series analysis, natural language processing, and audio processing. However, the simple RNN begins to forget earlier inputs and suffers from exploding gradients and vanishing gradients for large networks. In 1997, Hochreiter and Schmidhuber [79] proposed the long short-term memory to solve hard long time lag problems, and since then it has been successfully used in many sequence modeling tasks [80].

#### 2.3.2. LSTM

**Figure 2:**
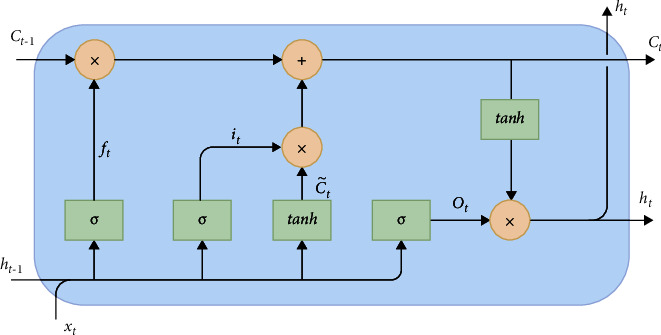
LSTM Architecture.

LSTM is a special kind of the RNN consisting of gates that can add or remove information from the cell state optionally [81].  Figure 2 illustrates the LSTM architecture.

LSTM consists of three types of gates: input gate, forget gate, and output gate [81]. The mathematics behind this is summarized in the following equations:

(4) $$\begin{array}{c} f_{t}=\sigma \left(W_{f}\cdot \left[h_{t-1},x_{t}\right]+b_{f}\right)\text{,}\\ i_{t}=\sigma \left(W_{i}\cdot \left[h_{t-1},x_{t}\right]+b_{i}\right)\text{,}\\ {\tilde{C}}_{t}=\tanh\;\left(W_{c}\cdot \left[h_{t-1},x_{t}\right]+b_{c}\right)\text{,}\\ C_{t}=f_{t}*C_{t-1}+i_{t}*{\tilde{C}}_{t}\text{,}\\ o_{t}=\sigma \left(W_{o}\cdot \left[h_{t-1},x_{t}\right]+b_{o}\right)\text{,}\\ h_{t}=o_{t}*\;\tanh\left(C_{t}\right)\text{,} \end{array}$$

where *f*_*t*_ is the forget gate that decides which information to pass through the cell state utilizing the sigmoid layer; *i*_*t*_ is the input gate that decides which input values will be added to the cell state; ${\tilde{C}}_{t}$ denotes the candidate values for the cell state; *C*_*t*_ is the new cell state value that is modified with the forget gate and the input gate; *o*_*t*_ is the output gate that decides the portion of the cell state activated with the hyperbolic tangent function; *h*_*t*_ is the cell state output from the cell state value and the decided output.

LSTM networks can be created with a single hidden layer or multiple hidden layers. The vanilla LSTM and stacked LSTM refer to a single hidden layer and multiple hidden layers, respectively. LSTM works well with sequence data in general, but some additional modifications are introduced to improve the results, such as peephole LSTM, bidirectional LSTM, and GRU.

#### 2.3.3. Bi-LSTM

**Figure 3:**
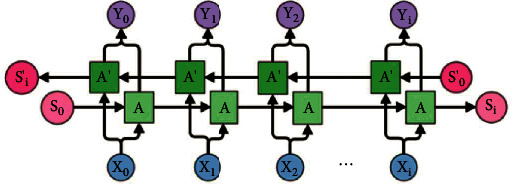
Bidirectional LSTM [82].

In essence, LSTM preserves information from inputs that have already passed through it using the hidden state. Unidirectional LSTM preserves information from the past because the only inputs it sees are from the past. Bidirectional LSTM (Bi-LSTM) uses inputs in two ways: one from the past to future and another from the future to past. The difference here is that in the LSTM that runs backwards, the information is preserved from the future and the usageof the two hidden states combined preserves information from both the past and the future [82]. In this way, the vanishing gradient problem can be solved. The flowchart of Bi-LSTM is depicted in Figure 3.

#### 2.3.4. GRU

**Figure 4:**
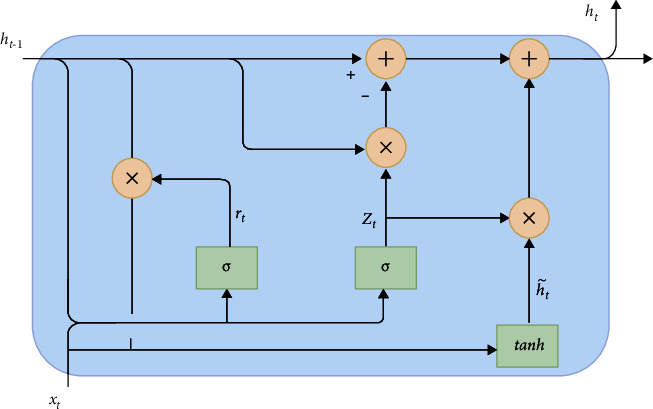
Gated recurrent unit.

The gated recurrent unit [83] is another variant of LSTM that has a different architecture. In this architecture, basically the input gate and forget gate are combined as a gate, which is called the update gate. The hidden state and cell state are concatenated for further simplification. The architecture of the GRU is illustrated in Figure 4.

Illustrated gates of the GRU can be formulated as given in the following equations:

(5) $$\begin{array}{c} z_{t}=\sigma \left(W_{z}\cdot \left[h_{t-1},x_{t}\right]\right)\text{,}\\ r_{t}=\sigma \left(W_{r}\cdot \left[h_{t-1},x_{t}\right]\right)\text{,}\\ {\tilde{h}}_{t}=\tanh\;\left(W\cdot \left[r_{t}*h_{t-1},x_{t}\right]\right)\text{,}\\ h_{t}=\left(1-z_{t}\right)*h_{t-1}+z_{t}*{\tilde{h}}_{t}\text{.} \end{array}$$

Here, *z*_*t*_ is the update gate that is used to determine how much information from the past should be passed to the next time instance; *r*_*t*_ indicates the reset gate that determines how much information from the past should be forgotten; ${\tilde{h}}_{t}$ and *h*_*t*_ are the candidate value and the new cell state value, respectively.

#### 2.3.5. CNN

**Figure 5:**
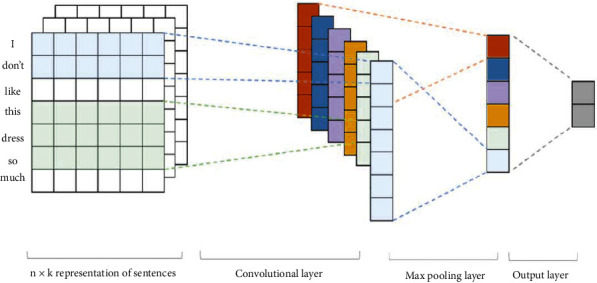
Text classification with the CNN.

The convolutional neural network (CNN) is a variant of traditional neural networks that are commonly used to analyze visual information [77]. In contrast to traditional neural networks, the CNN uses a convolution operation in order to extract features in hidden layers. Feature extraction with a convolution operation is a key to reducing more complex patterns into simpler patterns. The input of the CNN is generally an image, but instead of image pixels, the embedding matrix can also be used as an input for the CNN in order to perform natural language processing applications such as text classification and topic categorization [84, 85].  Figure 5 illustrates the architecture of the CNN that is used in text classification. Here, each row of the embedding matrix consists of a word vector. The convolution layer through the embedding matrix extracts N-gram features. Short-range and long-range relations can be extracted utilizing pooling layers [86].

## 3. Proposed Method

Recent advancements in technology facilitate to store and share big data on the internet, especially on social media platforms. Due to the expanding usage of social media, the volume of data on these platforms is rapidly increasing. New methods are required to be investigated to effectively analyze and interpret the big data. Modeling of the data with high volumes is a time-consuming process. Our main objective of this study is to propose a scalable method to cope with sentiment classification. In addition, we aim for the proposed method to be easily applicable without making extra training process for parameter or network optimization on such a large amount of data in different application areas. To do this, we propose a fuzzy rule-based inference system that is suitable for sentiment classification from the text data.

**Figure 6:**
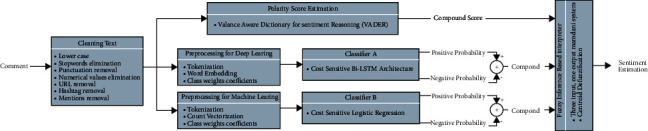
Block diagram of the proposed methodology.

The designed model is presented in Figure 6. The fuzzy inference mechanism is the last step of the model. In the design, the outputs of the pretrained models that successfully interpret the sentiments from the text are used as the inputs of the fuzzy inference system. The main idea here is to complete the final evaluation in a fuzzy rule system that combines the strengths of these methods.

In ensemble models, the similar problem is solved in general by assigning more weights to good classifiers or applying meta-learning approaches [87]. Meta-learning-based methods would require high times for parameter optimization, which may not be feasible in big data case. In the proposed method, we aim to improve the classification results by combining classical machine learning and deep learning models through a fuzzy inference mechanism to interpret the final evaluation considering the strength of each model.

**Figure 7:**
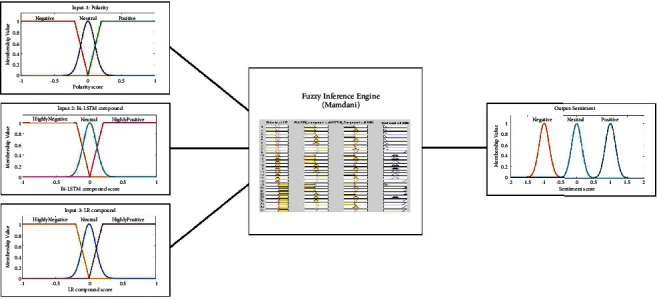
Proposed fuzzy inference mechanism.

The designed Mamdani-type fuzzy inference system is summarized in Figure 7. In this system, we defined three inputs and a single output. All the inputs are considered to indicate the compound score of sentiment that is expressed under the universe of discourse which is normalized to [−1,1]. The first input is named as the polarity score that is drawn from valance aware dictionary for sentiment reasoning. The second input is named as the compound score of the bidirectional-LSTM method. Long-short term memory-based methods are known to handle the vanishing gradient problem better than the classical methods [57]. The third input is the logistic regression compound score that is drawn by using cost sensitive logistic regression. The output is designed to return a value in [−1,1] to indicate the sentiment score. Here, approximately −1 indicates the negative sentiment, approximately 0 indicates the neutral sentiment, and approximately 1 indicates the positive sentiment for a 3-class sentiment classification. When the problem is a binary classification problem to detect if the sentiment is positive or negative, the sign of the defuzzified output, i.e., the final crisp value, is used as the class label. If the final crisp value is negative, then the test sample can be classified as a negative sentiment; otherwise, it can be classified as a positive sentiment.

Membership assignments and membership function (MF) parameters of the inputs and the output are presented in Table 1. Since all inputs are considered to be used in a normalized universe, we preferred to use trapezoidal membership functions for negative and positive subsets and Gaussian membership function for the neutral subset of each input. The output fuzzy sets are considered to be Gaussian membership functions for a smooth representation of approximate fuzzy numbers. Three inputs with three subsets and one output are connected with 27 fuzzy rules presented in Table 2, considering a 3-class sentiment classification. In this proposal, the membership assignments and rule definitions are designed intuitively in a very standard form. They can easily be modified, adopted, and used for different application areas by making corresponding assignments. For instance, in a binary classification problem, input/output membership functions can be modified in the form of two subsets as MF-1: negative and MF-2: positive, and the rules presented in Table 2 can be reduced to relate only the positive and negative aspects. Since the proposed method does not require retraining, it can also work well with big data.

## 4. Experimental Work

We performed four experiments to test the performance of the proposed method to compare it with some of the state-of-the-art methodologies in sentiment analysis. We used the following data sets in the test: (1) Coronavirus tweets NLP-text classification data set [88]; (2) Google Play application reviews [89]; (3) Amazon Alexa reviews [90]; (4) Rotten Tomatoes movies and critic reviews [91]. The experiment is considered as a 2-class sentiment classification problem if the tested data set has two class labels: positive and negative, or a 3-class sentiment classification problem if the tested data set has three class labels: positive, neutral, and negative.

The performance of the proposed methodology has been compared with both some famous classical machine learning methodologies such as logistic regression, support vector machines, and naive Bayes and some famous deep learning based methodologies such as the most popular versions of LSTM and GRU methods for each experiment. We applied the known methods with the most common and intensely used forms in the literature for sentiment analysis. We extracted features from comments by using term frequency-inverse document frequency and count vectorization for classical machine learning methods. For deep learning, we modeled and represented words in vector space using word embedding. In deep learning methodologies, we distributed class weights inversely proportional to the distribution of each class for balanced training. We used “He uniform” [92] as the weight initialization. In addition, if the model did not improve 5 epochs in a row, the learning rate would drop to one in 10 in order to converge better. The training is halted if it does not improve after 8 epochs. The hyperparameters used in model configuration for deep learning-based methods are batch size: 128; epochs: 15; cell size: 256; dropout: 0.2; learning rate: 0.01; decay rate: 0.1; optimizer: Adam; loss: categorical cross entropy. In order to compare the performances of the algorithms, we calculated the 3 × 3 confusion matrix of the 3-class classification problem and used the accuracy as the performance metric.

### 4.1. Experiment 1: Coronavirus-Tagged Data

Coronavirus tweets NLP-text classification data set [88] is a data set that is collected from tweets of people and manually tagged by the data set provider. Tweets reflect the opinion and emotions of people about the coronavirus disease. The data set is provided as the train and test sets separately from the data set owner. There are 41,159 observations in the training sets and 3,798 observations in the test sets. In the experiment, we considered the problem as a three-class classification problem from the text to sentiment. The fundamental three classes are negative, positive, and neutral classes. According to the training labels from the provider, 18,046 observations are tagged as the positive class; 15,398 observations are tagged as the negative class; 7,711 observations are considered as the neutral class. Experimental results are presented in Table 3.

Normalized correct recognition rates are shown in the columns of the table for each class. The value in the rightmost column corresponds to the 3-class overall classification accuracy. The results show that the proposed method gives the highest overall accuracy with 89%. It is followed by deep learning methods that give correct classification rates in the range of 83–85%. Naive Bayes has the lowest performance with the accuracy that is below 70%. Although the positive sentiment classification success of naive Bayes is the highest, a very bad neutral sentiment classification performance brings down the overall performance of the method. The proposed ensemble fuzzy method has the highest accuracy values in negative and neutral class classification and the second highest accuracy value in positive class classification. As a result, it produces a balanced high overall performance.

### 4.2. Experiment 2: Google Play Application Review Data

The second experiment is formed into a binary classification scheme to show that the proposed methodology can easily be adapted to other sentiment-related classification problems. The Google Play application review data set [89] includes three class labeled data inherently. In this experiment, we only considered the positive and negative classes. The membership functions have been used as identical to the previous case. The final sentiment decision has been made according to the sign of the defuzzified output by using centroid defuzzification. If the sign of the final crisp output of the defuzzification process is negative, it is labeled as the negative sentiment; if the sign of the final crisp output of the defuzzification process is positive, it is labeled as the positive sentiment. Experimental setup and parameter evaluation of the deep learning models are operated with the same framework as in the previous case. Experimental results are presented in Table 4.

In this experiment, we tested the 2-class sentiment performance of the methods. In general, all methods for this data set produced good performance values close to each other, within the range of 87–92%. The stacked GRU produced the highest rate in negative class correct recognition; CNN-LSTM produced the highest rate in positive class correct recognition; the proposed ensemble fuzzy method produced the highest rate in overall correct recognition accuracy. The method that is in the first place in the negative class has a low positive class performance, while the method that is in the first place in the positive class has a low negative class performance. Although the proposed method is the second highest one in both positive and negative class rankings, it ranks first in overall performance due to the close difference between both positive and negative class classification performance.

### 4.3. Experiment 3: Amazon Alexa Review Data

The Amazon Alexa data set [90] includes 3150 customer reviews and feedback for various Amazon Alexa products such as Echo, Echo dots, and Fire Stick. It consists of customer-verified reviews, ratings (stars), date of review, and variation. The data set is highly imbalanced with 409 negative and 2741 positive classes. We performed binary classification tests for this data set. The binary classification decision of the proposed method is given according to the sign of the defuzzified value obtained by applying centroid defuzzification of the final fuzzy output. If the sign is positive, the classification is considered as the positive class; if the sign is negative, the classification is considered as the negative class. Experimental results are presented in Table 5.

In this experiment, we obtained large differences between negative and positive class classification performances in many methods since the data set is unbalanced. Although SVM for TFIDF and count vectorization gave the highest overall accuracy, when negative and positive class performances are examined separately, we see that the performance of the SVM method is quite high in the positive class, which includes a much larger number of samples, and it is quite low in the negative class, which includes a much smaller number of samples. The overall classification performance of the SVM method is followed by the proposed fuzzy ensemble method and the naive Bayes method. For the naive Bayes method, there is a huge difference between negative and positive class classification performances. On the other hand, the positive and negative class classification performances of the proposed approach are much more balanced. As a result, although it does not give the highest performance, it can be said that the proposed fuzzy ensemble approach has a positive contribution to producing more balanced classification results, i.e., similar classification accuracies for both classes with large and small number of samples.

### 4.4. Experiment 4: Rotten Tomatoes Movies and Critic Review Data

Rotten Tomatoes website [93] is a movie news website that includes trailers, briefs, and critics. It is one of the most popular websites for movie reviews. The website presents a ranking called the Tomatometer that includes approved reviewers' comments and critics and an audience score that includes the percentage of users who rated the movie with 3.5 stars or higher. Approved Tomatometer critics make their final decision as “fresh” if their opinion is positive or “rotten” if their opinion is negative. The Rotten Tomatoes movies and critic review data set [91] is a large data set that has been created using the data scraped from the Rotten Tomatoes website. The data set consists of the movie data set, which contains information about more than seventeen thousand movies and the critic data set, which includes comments of critics. There is a total of 1,130,017 data in the data set. This data set contains much more negative sentiments than positive sentiments; i.e., it is also imbalanced. For this data set, we experimented with binary classification problems to infer “positive” and “negative” sentiments. Experimental results are presented in Table 6 in a similar form with the previous experiments.

The results in Table 6 show that the performance of the negative class with a much larger sample size is much higher than that of the positive class with a smaller sample number in logistic regression, SVM, and naive Bayes methods. There is a 20–44% difference in performance between positive class and negative class achievements of these methods. In deep learning-based approaches such as LSTM and GRU, the difference in correct classification between positive and negative classes is in the range of 5–11%. Although it is a lower rate than results of the classical methods, the difference is still large. On the other hand, the proposed method returned 83% correct classification rates for both positive and negative classes that makes an 83% overall classification accuracy. Although it does not give the highest performance in a single class, the overall recognition rate is high since there are no large performance differences between two classes with different sample sizes. The overall correct classification ratio of the proposed method is the highest rate among the tested methods in this data set.

### 4.5. Ablation Study

Ablation is known as the performance study of a multicomponent system by systematically removing specific components to understand their contribution to the overall system [94]. The fuzzy inference system proposed in this article produces the output based on 3 components, namely, polarity, Bi-LSTM, and logistic regression compounds. Here, we conducted an ablation study in this section to investigate the effects of these components on the overall system performance. In the study, we first killed two components at the input to obtain a single input-single output framework; then, we killed one component at each turn to obtain a two input-single output framework. At each turn, we determined the correct classification performances in all experiments that are given in the previous section.

#### 4.5.1. Part 1: Single Component Performance of the Proposed Method

**Figure 8:**
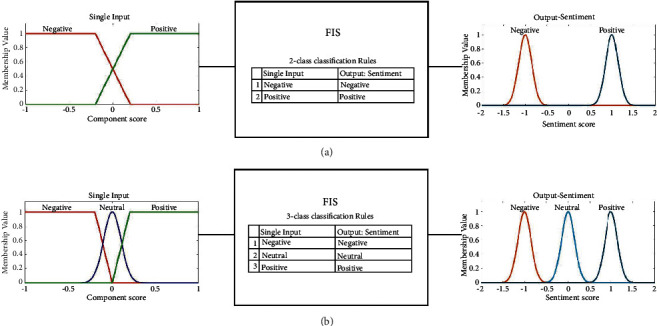
The fuzzy inference system used to test the single input-single output framework for the ablation study. (a) 2-class classification case. (b) 3-class classification case.

In this case, the proposed fuzzy inference system was reduced to a single input-single output framework. There are three components (input 1, 2, and 3), so we conducted three experiments for each single component in this part. We used the same fuzzy inference design parameters given in Table 1 with a reduced number of rules presented inTable 2 that were chosen based on the surviving components at the input. Note that some data sets in the experimental work are for 2-class and some are for 3-class classification.  Figure 8 summarizes the membership functions of the single input and the rules for both 2-class and 3-class classification cases.

#### 4.5.2. Part 2: Two-Component Performance of the Proposed Method

**Figure 9:**
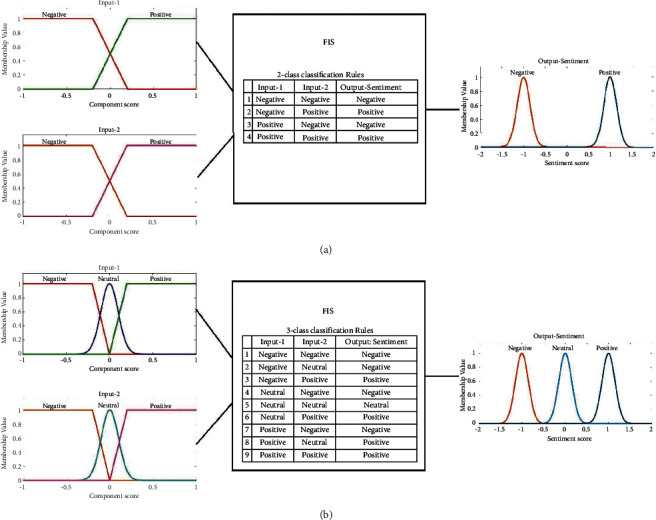
The fuzzy inference system used to test the single input-single output framework for the ablation study. (a) 2-class classification case. (b) 3-class classification case.

In this case, we killed one component and kept the other two components at the input that results in a two input-single output framework. We used the same fuzzy inference design parameters given in Table 1 with a reduced number of rules presented in Table 2 that were chosen based on the surviving components at the input. There are also 3 experiments for this case: (a) input 1: polarity; input 2: Bi-LSTM compound, (b) input 1: polarity; input 2: logistic regression compound, and (c) input 1: Bi-LSTM compound; input 2: logistic regression compound.  Figure 9 summarizes the membership functions and rules for the two input-single output framework for both binary and ternary classification cases.

#### 4.5.3. Part 3: Three-Component Performance of the Proposed Method

**Figure 10:**
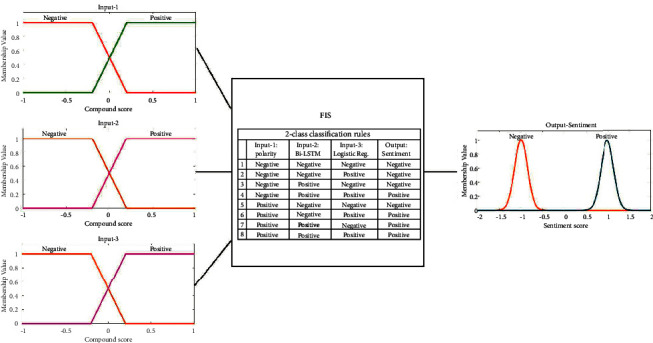
The binary classification framework of the proposed fuzzy inference mechanism.

Here, the proposed method was tested with a three input-single output framework. For the 3-class classification problem, it is used as shown in Figure 7 with the fuzzy inference design parameters given in Table 1. For the 2-class classification problem, it is used as summarized in Figure 10. Correct classification rates of the ablation study are given in Table 7.

**Figure 11:**
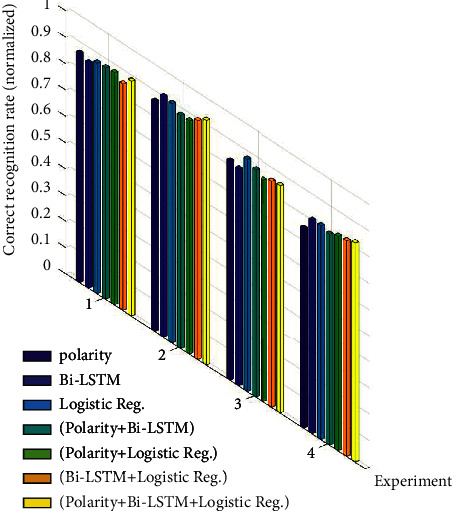
Bar plots of the ablation study. Correct classification rates are normalized in the plot.

The ablation study is illustrated in Figure 11 with the bar plot such that the horizontal axis represents the experiment number and the vertical axis represents the normalized overall classification accuracy values. Each bar corresponds to a correct classification rate obtained by killing some components and keeping others alive in the proposed method. In the figure, the components kept alive are shown with different colors. From left to right, the first three bars correspond to the presence of one component, the next three bars correspond to the presence of two components, and the rightmost yellow bar corresponds to the presence of 3 components, i.e., the proposed methodology.

Experiment 1 results show that one component and two-component cases give close results, but the three-component case produces the best accuracy. In the second data set, using all components still gives the best results, although some single-component cases produce slightly better values than some two-component cases. In the third data set, we see a different placement from the other experiments. Here, the presence of a single logistic regression component outperformed the other cases. The presence of three components performed in the second place. Experiment 3 is the Amazon Alexa review data set that consists of highly imbalanced sample sizes as mentioned. Since logistic regression alone produces much better results than other algorithms in this imbalance situation, when it is combined with other components, the overall performance drops slightly. Experiment 4 is the Rotten Tomatoes movies and critic review data set, which is the largest data set tested in this paper. Experiment 4 shows that the single-component case has a lower correct recognition rate in average, the two-component case has slightly higher values in average, and the three-component case has the highest accuracy value. As a result, the ablation study demonstrates that the proposed method produces more successful correct classification rates when used with 3 components as recommended.

## 5. Discussion

The 3-class sentiment performance of the proposed method was tested in the first experiment, and the 2-class sentiment performance of the proposed method was tested in other experiments. Experimental results show that the proposed ensemble fuzzy method gives better performance than the compared methods in most of the cases in terms of correct recognition rates for sentiment classification. It demonstrates that the designed fuzzy rule-based system integration has a positive effect on the performance of such important methods in sentiment classification.

The proposed method makes a combined evaluation by considering the results of three good sentiment analyzer components. We conducted an ablation study to determine how these components provide improvement in sentiment classification. The ablation study showed that evaluation of these three components when used together over the proposed fuzzy rule base increases the classification accuracy. It means that the proposed framework provides a good interpretation of sentiments.

Ensemble models usually concentrate on adjusting the proper weights of different methods to give overall good results. Even though the proposed fuzzy model is an ensemble model, there is no need of weight learning in our proposal. Inputs, outputs, and rule relations between them are defined through the fuzzy inference mechanism, and the final evaluation is a kind of a fuzzy logic process that is executed once. This type of intrinsic allows the proposed method to be used intuitively in other applications as well.

## 6. Conclusion

Sentiment analysis from the text is a compelling process since people's writing traditions, especially on social media, are not standard in terms of both writing styles and expressions. Therefore, it may not always be possible to find a method that gives the highest performance. In this article, we proposed an ensemble fuzzy inference system that performs sentiment analysis from the text by interpreting some current methods that yield very successful results in sentiment analysis through a fuzzy inference system. Unlike the classical ensemble methods, the proposed method not only allows weighting the base learners but also provides a way to combine the strengths of each algorithm via fuzzy rules. Although the proposed method has been tested with standard parameters, it gave more successful results than the other methods. Experimental work confirmed that the designed fuzzy rule-based system improved the classification performance in sentiment estimation. It may be possible to further increase the performance of the proposed method when the default parameters are tuned. It is also possible to extend the proposed method to be applied to different areas with different data sets. The training free nature of the proposed method makes it possible to be applied to large volumes of data in a similar manner. That is why, it would be more advantageous to use such a training-free method especially in platforms with constantly growing data volumes.

## Figures and Tables

**Table 1:** Designed fuzzy inference system variables and parameters.

Name	Input-1	Input-2	Input-3	Output
	Polarity	Bi-LSTM compound	Logistic regression compound	Sentiment
Range	[−1,1]	[−1,1]	[−1,1]	[−1,1]
MF-1 (negative)	Trapezoid, [−1, −1, −0.2,0]	Trapezoid, [−1, −1, −0.2,0]	Trapezoid, [−1, −1, −0.2,0]	Gaussian, *σ*=−1; *μ*=0.15
MF-2 (neutral)	Gaussian, *σ*=0; *μ*=0.1	Gaussian, *σ*=0; *μ*=0.1	Gaussian, *σ*=0; *μ*=0.1	Gaussian, *σ*=0; *μ*=0.15
MF-3 (positive)	Trapezoid, [0,0.2,1,1]	Trapezoid, [0,0.2,1,1]	Trapezoid, [0,0.2,1,1]	Gaussian, *σ*=1; *μ*=0.15

**Table 2:** Fuzzy rule list of the proposed fuzzy inference mechanism.

Rule	Inputs			Output
	Polarity	Bi-LSTM	Logistic regression	Sentiment
1	Negative	Negative	Negative	Negative
2	Negative	Negative	Neutral	Negative
3	Negative	Negative	Positive	Negative
4	Negative	Neutral	Negative	Negative
5	Negative	Neutral	Neutral	Negative
6	Negative	Neutral	Positive	Negative
7	Negative	Positive	Negative	Negative
8	Negative	Positive	Neutral	Negative
9	Negative	Positive	Positive	Positive
10	Neutral	Negative	Negative	Neutral
11	Neutral	Negative	Neutral	Neutral
12	Neutral	Negative	Positive	Neutral
13	Neutral	Neutral	Negative	Neutral
14	Neutral	Neutral	Neutral	Neutral
15	Neutral	Neutral	Positive	Neutral
16	Neutral	Positive	Negative	Neutral
17	Neutral	Positive	Neutral	Neutral
18	Neutral	Positive	Positive	Positive
19	Positive	Negative	Negative	Negative
20	Positive	Negative	Neutral	Positive
21	Positive	Negative	Positive	Positive
22	Positive	Neutral	Negative	Positive
23	Positive	Neutral	Neutral	Neutral
24	Positive	Neutral	Positive	Positive
25	Positive	Positive	Negative	Positive
26	Positive	Positive	Neutral	Positive
27	Positive	Positive	Positive	Positive

**Table 3:** Experimental results for coronavirus-tagged data set.

Method	Principle	Sentiment correct classification rates (normalized)			
		Negative sentiment	Neutral sentiment	Positive sentiment	3-class overall
Logistic regression	TFIDF	0.82	0.60	0.86	0.80
Cost sensitive logistic regression	TFIDF	0.79	0.76	0.78	0.78
Support vector machine	TFIDF	0.84	0.64	0.87	0.82
Naive Bayes	TFIDF	0.56	0.02	0.92	0.62
Logistic regression	Count vect.	0.82	0.72	0.85	0.82
Cost sensitive logistic regression	Count vect.	0.80	0.76	0.81	0.80
Support vector machine	Count vect.	0.81	0.69	0.85	0.81
Naive Bayes	Count vect.	0.76	0.13	0.79	0.67
Vanilla LSTM	Deep learning	0.81	0.73	0.88	0.83
Stacked LSTM	Deep learning	0.85	0.74	0.86	0.84
Bi-directional LSTM	Deep learning	0.86	0.81	0.83	0.84
GRU	Deep learning	0.85	0.76	0.87	0.84
Stacked GRU	Deep learning	0.87	0.77	0.87	0.85
CNN-LSTM	Deep learning	0.84	0.72	0.88	0.84
GRU-CNN	Deep learning	0.89	0.75	0.82	0.84
Proposed ensemble fuzzy method	Ensemble	0.90	0.87	0.88	0.89

**Table 4:** Experimental results for Google Play application review data.

Method	Principle	Sentiment correct classification rates (normalized)		
		Negative sentiment	Positive sentiment	Overall
Logistic regression	TFIDF	0.88	0.90	0.89
Cost sensitive logistic regression	TFIDF	0.89	0.89	0.89
Support vector machine	TFIDF	0.91	0.91	0.91
Naive Bayes	TFIDF	0.85	0.91	0.88
Logistic regression	Count vect	0.89	0.92	0.91
Cost sensitive logistic regression	Count vect	0.90	0.91	0.91
Support vector machine	Count vect	0.89	0.92	0.91
Naive Bayes	Count vect	0.86	0.90	0.88
Vanilla LSTM	Deep learning	0.90	0.84	0.87
Stacked LSTM	Deep learning	0.85	0.88	0.87
Bi-directional LSTM	Deep learning	0.89	0.92	0.91
GRU	Deep learning	0.87	0.91	0.89
Stacked GRU	Deep learning	0.92	0.85	0.88
CNN-LSTM	Deep learning	0.83	0.95	0.89
GRU-CNN	Deep learning	0.86	0.92	0.89
Proposed ensemble fuzzy method	Ensemble	0.91	0.93	0.92

**Table 5:** Experimental results for Amazon Alexa review data.

Method	Principle	Sentiment correct classification rates (normalized)		
		Negative sentiment	Positive sentiment	Overall
Logistic regression	TFIDF	0.06	1.00	0.88
Cost sensitive logistic regression	TFIDF	0.83	0.88	0.87
Support vector machine	TFIDF	0.47	0.98	0.91
Naive Bayes	TFIDF	0.01	1.00	0.87
Logistic regression	Count vect	0.06	1.00	0.88
Cost sensitive logistic regression	Count vect	0.83	0.88	0.87
Support vector machine	Count vect	0.47	0.98	0.91
Naive Bayes	Count vect	0.01	1.00	0.87
Vanilla LSTM	Deep learning	0.78	0.79	0.79
Stacked LSTM	Deep learning	0.64	0.83	0.81
Bi-directional LSTM	Deep learning	0.78	0.79	0.79
GRU	Deep learning	0.81	0.84	0.84
Stacked GRU	Deep learning	0.80	0.84	0.83
CNN-LSTM	Deep learning	0.60	0.90	0.86
GRU-CNN	Deep learning	0.70	0.88	0.86
Proposed ensemble fuzzy method	Ensemble	0.90	0.86	0.87

**Table 6:** Experimental results for Rotten Tomatoes movies and critic review data.

Method	Principle	Sentiment correct classification rates (normalized)		
		Negative sentiment	Positive sentiment	Overall
Logistic regression	TFIDF	0.89	0.68	0.82
Cost sensitive logistic regression	TFIDF	0.81	0.80	0.81
Support vector machine	TFIDF	0.88	0.70	0.81
Naive Bayes	TFIDF	0.94	0.50	0.78
Logistic regression	Count vect	0.89	0.69	0.81
Cost sensitive logistic regression	Count vect	0.81	0.80	0.81
Support vector machine	Count vect	0.88	0.69	0.81
Naive Bayes	Count vect	0.85	0.72	0.80
Vanilla LSTM	Deep learning	0.84	0.73	0.77
Stacked LSTM	Deep learning	0.84	0.74	0.77
Bi-directional LSTM	Deep learning	0.85	0.74	0.78
GRU	Deep learning	0.83	0.74	0.77
Stacked GRU	Deep learning	0.84	0.74	0.77
CNN-LSTM	Deep learning	0.79	0.74	0.76
GRU-CNN	Deep learning	0.78	0.74	0.76
Proposed ensemble fuzzy method	Ensemble	0.83	0.83	0.83

**Table 7:** Correct classification rates (in %) of the ablation study.

Experiment	Single component			Two components			Three components
		Polarity	Bi-LSTM	Logistic reg	(Polarity + Bi-LSTM)	(Polarity + logistic reg)	(Bi-LSTM + logistic reg)	Proposed framework
Experiment 1: Coronavirus-tagged data	3-class	87.15	85.57	87.67	87.94	88.20	86.15	89.31
Experiment 2: Google Play reviews	2-class	87.44	91.33	90.83	88.40	88.43	90.61	92.87
Experiment 3: Amazon Alexa	2-class	83.27	82.33	88.19	86.27	84.56	86.23	86.72
Experiment 4: Rotten Tomatoes	2-class	76.28	81.47	81.53	80.41	81.77	82.15	83.48

## Data Availability

Publicly available datasets are deposited in a repository. These prior datasets are cited at relevant places within the text as references. There are four experiments in our study, and each uses a popular dataset on sentiment analysis that are previously shared by their creators in kaggle repository. Corresponding webpages of them are given as references, and if they recommend a paper citation, the papers are also cited in the text properly.
